# ALT-803 in the treatment of non-muscle-invasive bladder cancer: Preclinical and clinical evidence and translational potential

**DOI:** 10.3389/fimmu.2022.1040669

**Published:** 2022-11-10

**Authors:** Wujun Chen, Ning Liu, Yang Yuan, Meng Zhu, Xiaokun Hu, Wenchao Hu, Shuai Wang, Chao Wang, Binghuan Huang, Dongming Xing

**Affiliations:** ^1^ Cancer Institute, The Affiliated Hospital of Qingdao University, Qingdao University, Qingdao Cancer Institute, Qingdao, Shandong, China; ^2^ Department of Neurosurgery, The Affiliated Hospital of Qingdao University, Qingdao, Shandong, China; ^3^ Interventional Medicine Center, The Affiliated Hospital of Qingdao University, Qingdao, Shandong, China; ^4^ Department of Endocrinology, Qilu Hospital (Qingdao), Cheeloo College of Medicine, Shandong University, Qingdao, Shandong, China; ^5^ School of Medical Imaging, Radiotherapy Department, Affiliated Hospital of Weifang Medical University, Weifang Medical University, Weifang, Shandong, China; ^6^ School of Life Sciences, Tsinghua University, Beijing, China

**Keywords:** NMIBC, ALT-803, pembrolizumab, oportuzumab monatox, BCG

## Abstract

Bladder cancer (BCa) is one of the most common malignant tumors that cause death. Approximately 75%–85% of BCa develop into non-muscle-invasive bladder cancer (NMIBC). Bacillus Calmette-Guérin (BCG) is the gold standard for avoiding cystectomy in the treatment of NMIBC. Unfortunately, up to 30% of patients do not respond to BCG treatment, and up to 70% of BCG responders relapse. The United States Food and Drug Administration (FDA) approved valrubicin (1998) and pembrolizumab (2020) for the treatment of BCG-unresponsive (BCGu) NMBIC. However, the complete remission rate for valrubicin and pembrolizumab was only 16% and 40.6%, respectively. ALT-803 (N-803) is an IL-15 superagonist and reduces tumor burden by promoting the proliferation and activation of NK cells and CD8^+^ T cells. The FDA received (23 May 2022) and accepted to review (28 July 2022) the marketing submission of ALT-803 plus BCG for the treatment of BCGu NMIBC. However, the FDA previously rejected the application for oportuzumab monatox (OM) due to a lack of data comparing it with pembrolizumab on August 20, 2021. Interestingly, the clinical efficacy and safety of ALT-803 were higher than that of pembrolizumab and OM, suggesting that ALT-803 may be approved by FDA. This review aims to further knowledge of the preclinical and clinical evidence of ALT-803 in the treatment of NMIBC and discuss its translational potential.

## 1 Introduction

Bladder cancer (BCa) is the 10th most common cancer in the world and the 11th leading cause of death among solid tumors. It includes muscle-invasive bladder cancer (MIBC) and non-muscle-invasive bladder cancer (NMIBC), with the latter accounting for approximately 75% to 85% of cases. NMIBC includes tumors infiltrating the lamina propria (T1), carcinomas *in situ* (CIS), and papillary noninvasive carcinomas (Ta). Radical cystectomy is the gold standard in the treatment of high-risk NMIBC. However, many patients with NMIBC are unwilling or unable to undergo surgery due to the consequent poor quality of life. As of August 2022, the United States Food and Drug Administration (FDA) has approved only Bacillus Calmette-Guérin (BCG) (in 1976), valrubicin (in 1998), and pembrolizumab (in 2020) for the treatment of NMIBC (1–4). BCG is a live attenuated bovine tuberculosis bacillus. Intravesical BCG is used for the treatment of intermediate- and high-risk NMIBC as adjuvant therapy. BCG can prolong the progression-free interval after initial tumor resection in patients with NMIBC. It exhibits a nonspecific immune response to reduce tumor burden by increasing the number of natural killer (NK) cells and CD8^+^ T cells and by cytokine secretion, including that of interleukin (IL)-2, IL-12, IL-18, and interferon-gamma (INF-γ) (5, 6). However, there are no biomarkers for predicting the response to BCG. Furthermore, the risk of NMIBC tumor recurrence is very high (~70%), and many patients experience relapses and eventually develop resistance to BCG (*i*.*e*., they show no response) (6, 7). Such patients have to undergo cystectomy, which has a 9% mortality rate, have a poor quality of life, and bear high costs. Valrubicin is the first drug approved by the FDA to treat BCG-unresponsive (BCGu) NMIBC. However, the response rate for valrubicin was only 16%. In response to this difficult-to-treat disease, the FDA approved pembrolizumab for the treatment of BCGu NMIBC based on phase 2 clinical data. However, the complete response (CR) rate of pembrolizumab was only 40.6% in the phase 2 trial (8). Therefore, new therapies are urgently needed to prevent disease progression and allow bladder retention to maintain the patient’s quality of life.

IL-15, which is a 15-kDa cytokine, is a four-helix bundle common gamma chain cytokine and a promising target for cancer immunotherapy. The US National Cancer Institute listed IL-15 as the drug target with the greatest potential for cancer immunotherapy in 2008 (9). IL-15 is a potent stimulator of CD8^+^ T cells and NK cells *via* the activation of IL-15Rα, IL-2Rβ (also named CD122), and IL-2Rγ (also named CD132), which share receptors with IL-2, IL-4, IL-7, IL-9, and IL-21, and thereby reduces tumor burden (10, 11). ALT-803 [also named N-803 (nogapendekin alfa inbakicept), Anktiva, and IL-15SA], which is an IL-15 cytokine antibody fusion protein, is a complex of the IL15 superagonist N72D mutant and the dimer IL15 receptor α Su/IgG1 Fc fusion protein. ALT-803 enhances the tumor killing activity of NK and CD8^+^ T cells and has a longer half-life than recombinant IL-15, leading to a potent antitumor effect (12). The efficacy, safety, and tolerability of ALT-803 alone or in combination with BCG for the treatment of NMIBC were good in preclinical (utilizing animal models) and clinical studies (phases 1–3) (13, 14). The FDA awarded ALT-803 fast track designation and breakthrough designation status in 2017 and 2019, respectively (14, 15). ALT-803 was originally developed by Altor Biosciences and is currently being developed by ImmunityBio. Inc. for the treatment of NMBIC. ImmunityBio. Inc. submitted a marketing submission to the FDA for ALT-803 in combination with BCG for the treatment of BCGu NMIBC CIS with or without Ta or T1 disease on 23 May 2022. The FDA accepted to review this application on 28 July 2022, and the Prescription Drug User Fee Act target action date is 23 May 2023. However, whether it will be approved is a moot point according to the news reported on the website of ImmunityBio. Inc. The FDA previously rejected the marketing application for oportuzumab monatox (OM; also named as VIcineum, Vicinium, Vysyneum, and VB4-845) by Sesen Bio. Inc. due to a lack of data comparing it with pembrolizumab or other chemotherapeutics on 23 August 2021. The EU marketing authorization application of OM was withdrawn on 27 August 2021 (16, 17). Indeed Sesen Bio. Inc. plans to resolve the issue with OM and apply for approval again. ALT-803 may also require a phase 3 trial that compares it with pembrolizumab (18–20). In this review, we focus on the advances in the development of ALT-803 for the treatment of NMIBC at the preclinical and clinical stages to discuss its translational potential from the perspectives of pharmacology, pharmacokinetics, and toxicology.

## 2 The preclinical evidence of using ALT-803 in NMIBC

### 2.1 Pharmacology

#### 2.1.1 Normal C57BL/6J mice

ALT-803 shows strong immunity in normal C57BL/6J mice. Subcutaneous (S.C.) and intravenous (I.V.) ALT-803 promote the serum IL-6, IFNγ, MCP-1, granzyme B (GzB), and TNFα levels but do not change the IL-10 and IL-12 levels in C57BL/6 mice. ALT-803 also promotes IFNγ and TNFα secretion but does not change the IL-6, IL-2, IL-10, IL-4, and IL-17A levels in mouse splenocytes (21). MCP-1 plays a crucial role in enhancing monocyte migration and infiltration, T cell number, and NK cell memory. Meanwhile, GzB plays a key role in NK cell cytotoxicity (22). These findings suggest that ALT-803 exhibits anti-tumor activity by enhancing the MCP-1 and GzB expression.

**Figure 1:**
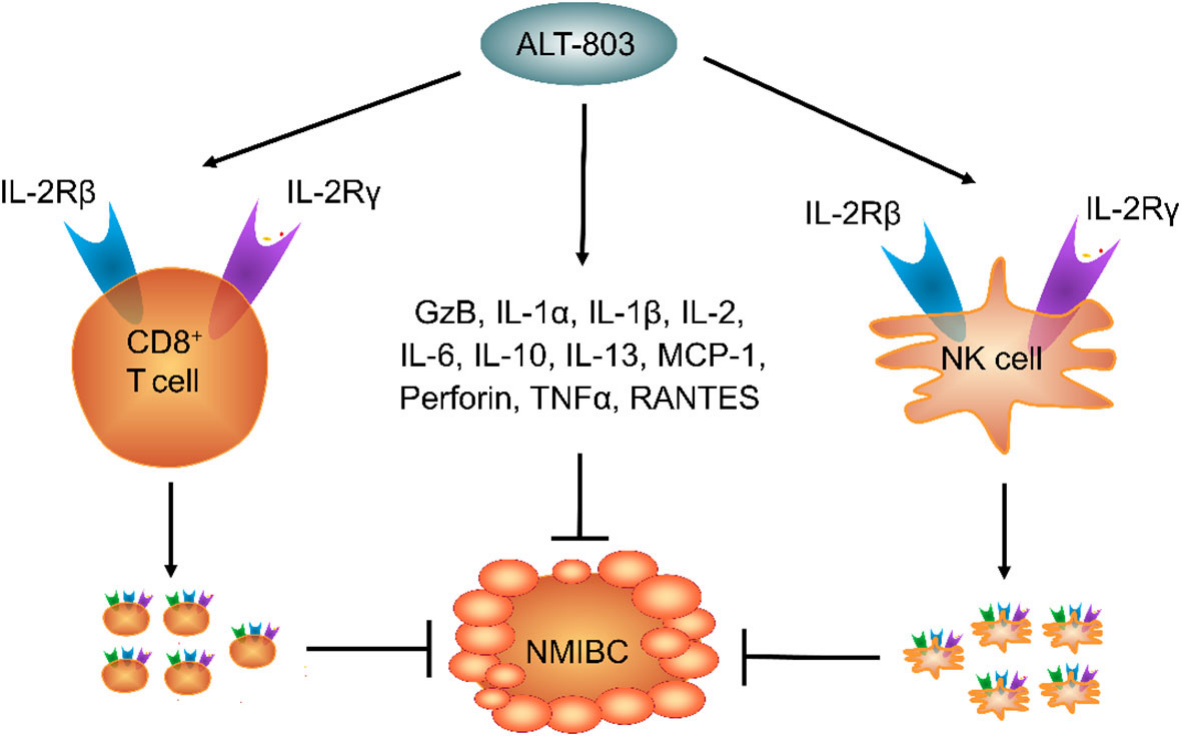
The mechanism of ALT-803 for the treatment of non-muscle-invasive bladder cancer.

ALT-803 also stimulates immune cell infiltration into the lymphoid organs in a dose-dependent manner in C57BL/6 mice. ALT-803, but not recombinant IL-15 (rIL-15), increases the spleen weight and the CD8^+^ T cell and NK cell counts in C57BL/6 mice, suggesting that the immune efficacy of ALT-803 is higher than that of rIL-15 (21, 22). Interestingly, the effect of S.C. ALT-803 on CD8^+^ T, Treg, and NK cell proliferation in the spleen is similar to that of I.V. ALT-803 in C57BL/6 mice. The S.C. ALT-803-mediated upregulation of GzB expression is also similar to that observed after I.V. treatment, suggesting that both S.C. and I.V. ALT-803 have similar effects on NK cell cytotoxicity (21, 22). Many studies have shown that the immune efficacy of ALT-803 in normal animals is similar to that in tumor animal models, suggesting that ALT-803 suppresses cancer development by enhancing the activity of CD8^+^ T and NK cells and the expression of MCP-1 and GzB (**Figure 1**).

#### 2.1.2 N-Butyl-N-(4-hydroxybutyl) nitrosamine-induced C57BL/6J mice orthotopic NMIBC model

N-Butyl-N-(4-hydroxybutyl) nitrosamine (BBN) is a highly carcinogenic compound and induces orthotopic NMIBC. In a previous study involving this carcinogen-induced mouse NMIBC model, S.C. ALT-803 was found to reduce the tumor burden by 37%, which is a higher reduction than that obtained with intravesical ALT-803 (28%) or intravesical BCG (28%), suggesting that the efficacy of S.C. ALT-803 is better than that of intravesical ALT-803 or BCG (13). Although the efficacy of intravesical ALT-803 is similar to that of intravesical BCG, intravesical ALT-803 tends to outperform intravesical BCG. Additionally, S.C. or intravesical ALT-803 combined with intravesical BCG reduces tumor burden by 44% and 36% in this model, respectively, suggesting that ALT-803 enhances the efficacy of BCG. Furthermore, S.C. ALT-803 increases the population of peripheral CD8^+^ T, NK (NKG2D^+^), NKT (CD3^+^/NKG2D^+^), and splenic tissue NKT cells. However, intravesical ALT-803 alone and intravesical BCG alone did not affect these lymphocytes, suggesting that the systemic effects obtained with S.C. ALT-803 are higher than that with intravesical ALT-803 and BCG. Additionally, intravesical BCG suppresses the S.C. ALT-803-mediated induction of lymphocyte population but increases the peripheral CD8^+^ T cell population by intravesical ALT-803, suggesting that intravesical ALT-803 combined with intravesical BCG has little to no systemic effects (13).

S.C. ALT-803 also regulates more cytokines than intravesical ALT-803 and BCG. Specifically, S.C. ALT-803 was found to increase the sera IL-5 and IL-6 levels and urine IL-13 levels, and the increase was higher than that obtained with intravesical BCG. Furthermore, S.C. ALT-803 combined with intravesical BCG was found to increase the sera TNFα, IL-5, IL-6, and IL-13 levels and the urine IL-13 and IFN levels. S.C. ALT-803 alone, S.C., or intravesical ALT-803 combined with intravesical BCG reduces the sera IL-1β level. However, intravesical ALT-803 combined with intravesical BCG does not affect any urine cytokines, suggesting that S.C. ALT-803 elicits a systemic immune response compared to intravesical ALT-803 (13). Taken together, S.C. ALT-803 alone or in combination with intravesical BCG was found to exhibit a potent antitumor effect compared to intravesical ALT-803 alone or BCG alone or their combined administration. S.C. administration may be the most advantageous approach due to its stronger systemic immune response than intravesical administration. However, more clinical trial studies are needed.

#### 2.1.3 BBN-induced SD rat orthotopic NMIBC model

Preclinical studies with multiple animal models are a prerequisite for the development of new drugs and subsequent clinical applications. Intravesical ALT-803 alone or in combination with intravesical BCG was reported to reduce the tumor burden by 35% and 46%, respectively, by enhancing NK cell activation in the BBN-induced SD rat orthotopic NMIBC model and was more effective than intravesical BCG alone (15%). The combination treatment reduced the tumor angiogenesis and proliferation of the tumor cells by 76% and 80%, respectively, which is higher than that observed with intravesical ALT-803 alone (59% and 52%) and intravesical BCG alone (40% and 40%) (23). These findings suggest the efficacy of intravesical ALT-803 alone or in combination with intravesical BCG than that of intravesical BGC alone in rats.

Intravesical ALT-803 alone and in combination with BCG was reported to increase the number of tumoral microenvironment CD3^+^ T cells but not of that found in the blood and spleen. Furthermore, the increase was higher than that obtained with intravesical BCG alone. The combination of ALT-803 and BCG enhanced the tumoral NK cell numbers to a higher extent than intravesical ALT-803 alone or BCG alone. Intravesical ALT-803 alone, intravesical BCG alone, and the combination of ALT-803 and BCG promoted the number of tumoral CD8^+^ T cells and NK cells in the blood and spleen (23). However, there was no statistical significance among the groups, suggesting that intravesical ALT-803 in combination with BCG reduced the tumor burden of bladder cancer by increasing the infiltration and cytotoxicity of CD3^+^ T cells and NK cells in the tumor tissue.

IL-1α and IL-1β are secreted by macrophages and neutrophils. RANTES (also named CCL5) enhances antitumor immunity through the recruitment and activation of NK cells. Intravesical ALT-803 was found to enhance the proliferation and activation of NK cells by stimulating the secretion of IL-1α, IL-1β, and RANTES, which are Th1 cytokines. Notably, the levels of urinary Th1 cytokines (such as IFNγ, IL-2, and IL-12) were reported to be positively correlated with the BCG response, while the levels of Th2 cytokines (such as IL-10 and IL-6) showed a negative correlation (23). Based on the synergistic effect of ALT-803 and BCG, we hypothesize that Th1 cytokines may play a key role in the efficacy of intravesical ALT-803.

### 2.2 Pharmacokinetics

#### 2.2.1 Mice

The hepatobiliary pathway is the dominant mechanism for clearing ALT-803, whereas the renal pathway mainly functions for clearing rIL-15 (21). ALT-803 has a serum half-life of 7.50 h after I.V. injection and 7.71 h after S.C. injection in C57BL/6 mice, suggesting that the half-life of I.V. ALT-803 is similar to that of S.C. ALT-803. The half-life of I.V. ALT-803 evaluated using anti-IL-15 Ab and anti-IgG Fc Ab was 25 and 18 h in ICR (CD-1) mice, respectively, suggesting that ALT-803 has a long half-life. Indeed rIL-15 requires daily or continuous intravenous administration due to poor bioavailability in lymphoid tissues and rapid clearance. In contrast, ALT-803 induces maximum immune cell stimulation 4 days after administration, suggesting that ALT-803 can be administered clinically once or twice a week (21, 22).

The maximum serum concentration (C_max_) of I.V. ALT-803 (0.2 mg/kg) in C57BL/6 mice is 3,926 ng/ml at 0.5 h, whereas that of S.C. injection (0.2 mg/kg) is 495 ng/ml at 16 h, suggesting that the C_max_ of I.V. administration is higher than that of S.C. administration. I.V. administration takes less time to achieve efficacy than S.C. administration. However, the side effects of I.V. administration may be more than that of S.C. administration as I.V. administration would lead to higher serum levels (22).

The tissue distribution and residence time of I.V. and S.C. ALT-803 in lymphoid tissues are higher than that of rIL-15, suggesting that ALT-803 not only has a longer serum half-life but also has a higher distribution and residence time in the lymphoid organs than rIL15. Specifically, ALT-803 level has been detected in a variety of tissues in female C57BL/6NHsd (B6) mice and C57BL/6 mice, including in the spleen, lymph nodes, bone, liver, stomach, lung, heart, kidney, blood, brain, intestine, muscle, pancreas, thymus, skin, and tail, whereas IL-15 level by I.V. rIL-15 was detected only in the liver, kidney, and lymph nodes, with very low levels observed in other organs (21, 22). Moreover, the ALT-803 levels by S.C. administration in the spleen, lymph nodes, bone, and kidney were higher than that by I.V. administration, whereas its level in the liver, lung, stomach, intestine, and heart was lower than that by I.V. administration, suggesting that S.C. administration may have high immune stimulation than I.V. administration although I.V. administration has a higher C_max_ (21, 22). Taken together, ALT-803 has a long blood circulation time and low kidney uptake and accumulates over a wide area and for a long time in multiple tissues.

#### 2.2.2 Monkeys

ALT-803 also has a longer half-life in monkeys. I.V. ALT-803 (0.1 and 0.03 mg/kg) showed a non-dose-dependent plasma half-life (7.2–8 h) in cynomolgus monkeys. The plasma C_max_ of 0.1 mg/kg ALT-803 was 30 nmol/L in cynomolgus monkeys, whereas the C_max_ of 0.03 mg/kg ALT-803 was 6 nmol/L and enhanced the immune cell proliferation and activation by 60-fold compared to that *in vitro* (concentration of 0.1 nmol/L). These data suggest that a low dose could enhance immune cell proliferation and activation. However, I.V. rIL-15 (10, 20, and 50 μg/kg/day) has a short plasma half-life ranging from 0.907 to 2.159 h in rhesus macaques and macaques. The C_max_ of IL-15 by I.V. rIL-15 ranged from 450.7 to 2,085 ng/ml in rhesus macaques, suggesting that the half-life and efficacy of ALT-803 are higher than that of rIL-15 in monkeys. ALT-803 also increases the count of white blood cells (WBCs; such as monocytes) and lymphocytes [such as NK cells, CD4^+^ T cell (including naïve, central memory, and effector memory subtype), and CD8^+^ T cells] in a dose-dependent manner in monkeys as well as the lymphocytic infiltration and hyperplasia in the liver, kidneys, and lungs. However, ALT-803 did not affect the blood IFNγ, TNFα, IL-6, IL-5, IL-4, and IL-2 levels, suggesting that ALT-803 does not trigger a cytokine storm in monkeys (21, 24, 25).

### 2.3 Toxicology

#### 2.3.1 Murine

##### 2.3.1.1 S.C. ALT-803

S.C. ALT-803 has no dose-limiting toxicity in B6 mice. A high dose of ALT-803 (1.0 mg/kg/week, S.C., 4 weeks) did not have obvious side effects in female B6 mice (21, 22). There was a trend of weight loss and side effects of arching at the beginning of the treatment. However, these side effects disappeared as the treatment progressed. S.C. ALT-803 reduced the blood alkaline phosphatase (ALP) and creatinine levels but did not affect the aspartate, alanine aminotransferases, and blood urea nitrogen levels, suggesting that ALT-803 shows no hepatotoxicity and nephrotoxicity (21, 22). Phosphate ester prodrugs (such as etoposide phosphate and fosamprenavir) are rapidly cleaved into the active compound by human ALP (26), suggesting that ALT-803 cannot be combined with phosphate ester prodrugs.

S.C. ALT-803 altered the weight and hematology of the tissues, including the weight of the spleen (by 5.5-fold), lymph node (by threefold), and the liver (by approximately 0.2-fold), enhancing erythropoiesis to leukocyte conversion and multiple cell counts such as that of WBCs (by ninefold), lymphocytes (by ninefold), neutrophils (by eightfold), monocytes (by sevenfold), eosinophils (by sixfold), and basophils (by fourfold). The liver tissue contains many NK cells, suggesting that the ALT-803-induced increase in the liver weight may be due to ALT-803-mediated NK cell proliferation and activation (21, 22).

##### 2.3.1.2 I.V. ALT-803

A high dose of I.V. ALT-803 promoted weight loss and death in C57BL/6 mice. The dead mice presented with pulmonary edema and enlarged lymph nodes and spleen. I.V. ALT-803 also increased the tissue weights and hematological counts, which were higher than those obtained with S.C. ALT-803 [spleen (by 6.5-fold), WBCs (by 15-fold), lymphocytes (by 18-fold), neutrophils (by 10-fold), monocytes (by 11-fold), eosinophils (by sevenfold), and basophils (by sixfold) (21, 22). Cytokines promote these symptoms by inducing inflammatory responses, suggesting that a high dose of ALT-803 induced systemic inflammatory responses. Taken together, I.V. ALT-803 may show systemic toxicity due to its higher ALT-803 peak serum concentration, which could trigger higher inflammatory cell counts and a cytokine storm.

##### 2.3.1.3 Intravesical ALT-803

Intravesical ALT-803 has no dose-limiting toxicity in SD rats. Intravesical ALT-803 alone or in combination with BCG is safe and well tolerated (23).

#### 2.3.2 Monkeys

I.V. ALT-803 was reported to induce liver necrosis and bone marrow hyperplasia in monkeys. Indeed I.V. IL15:IL15Rα/Fc complex (0.5 mg/kg/4 days) promoted mice death by enhancing acute hepatocellular injury by NK cells, suggesting that I.V. ALT-803 may cause liver injury in clinical practice. Additionally, I.V. ALT-803 reduced the serum albumin but did not affect the serum levels of liver enzymes in monkeys (21). Albumin is the main protein causing hypoalbuminemia. Furthermore, the serum-albumin-to-globulin ratio levels can independently predict the risk of disease progression in patients with NMIBC (27). Thus, one must be wary of the risk of hypoproteinemia by I.V. ALT-803 when used for treating NMIBC. Meanwhile, the side effects of I.V. rIL-15 include loss of appetite, diarrhea, vomiting, weight loss (ranging from 11% to 12%), high levels of triglycerides, and low levels of phosphorus in rhesus macaques. rIL-15 also promotes the enlargement of the liver (lymphocytes and neutrophils) and spleen, bone marrow hyperplasia, loss of adipocyte number, pulmonary edema, and moderate thrombosis in rhesus macaques (24, 25). As LT-803 and rIL-15 have the same target, one should consider these side effects when administering I.V. ALT-803. Based on the pharmacokinetic (PK) profiles and safety of ALT-803 in mice and monkeys, S.C. ALT-803 is superior to I.V. ALT-803.

Indeed there are no adverse effects of I.V. 0.1 mg/kg ALT-803 in mice and I.V. 0.03 mg/kg ALT-803 in cynomolgus monkeys, suggesting that approximately 10 μg/kg ALT-803 is reasonable for human administration according to the efficacy conversion factors from animal to human. In addition, the serum C_max_ of ALT-803 after 5 μg/kg administration is 1 nmo/L, which is a sufficient dose to promote the proliferation and activation of human T and NK cells *in vitro*. Based on the therapeutic dose window of ALT-803 results, a dose of 1.0 to 5.0 mg/kg ALT-803 in humans may be sufficient to stimulate immune cells and have no toxicologic effects (21).

## 3 The clinical evidence of using ALT-803 in NMIBC

### 3.1 Pharmacology

#### 3.1.1 *In vitro*

ALT-803 shows strong immune efficacy in human cells *in vitro*. Specifically, ALT-803 was found to increase the lymphocyte counts (by 2.1-fold) in 7-day cultures of human peripheral blood mononuclear cells (PBMCs) *in vitro*, including that of CD8^+^ T (by 3.0-fold), CD4^+^ T (by 1.8-fold), and NK (by 2.8-fold) cells, and exhibit nanomolar efficacy (0.5 nM). ALT-803 also increased the cell surface activation marker CD69 (NK and CD8^+^ T cells) and CD25 expression (NK and CD4^+^ T cells) in a dose-dependent manner (21). GzB and perforin play a key role in the cytotoxic activity of NK cells and CD8^+^ T cells on tumor cells. ALT-803 increased the expression of GzB and perforin in NK cells and CD8^+^ T cells in a dose-dependent manner, suggesting that ALT-803 kills tumor cells by enhancing GzB and perforin expression and the subsequent activation of NK and CD8^+^ T cells (21). Indeed 0.07 nM ALT-803 can increase the CD8^+^ T cell population by more than 1.5-fold, and 0.01 nM ALT-803 can promote GzB and perforin expression in NK cells and CD8^+^ T cells, suggesting that ALT-803 has strong immune efficacy. In addition, ALT-803 promotes IFNγ and IL-6 secretion but does not alter the levels of TNFα, IL-4, IL-10, and IL-17A in human PBMCs (21). As mentioned above, ALT-803 also promotes IFNγ expression and lymphocyte proliferation in mice, suggesting that IFNγ and lymphocytes play a key role in the immune efficacy of ALT-803 in mice as well as humans.

#### 3.1.2 Phase 1 trial (healthy human volunteers)

S.C. ALT-803 demonstrated a strong immune effect and good tolerance in healthy human volunteers (*N* = 20). Specifically, S.C. ALT-803 promoted the proliferation of NK cells, CD8^+^ T cells, CD4^+^ T cells, and γδ T cells from the PBMCs of volunteers, particularly NK cells. S.C. ALT-803 also promotes NK cells and CD8^+^ T cells to kill tumor cells by enhancing NKG2D, NKp30, and GzB expression. S.C. ALT-803 promotes the serum IFN-γ, IL-6, and IL-10 levels but does not change the TNFα, IL-4, and IL-2 levels in these healthy volunteers. The immunological effect of S.C. ALT-803 in healthy human volunteers is similar to that observed in patients with cancer, suggesting that S.C. ALT-803 may have the potential to inhibit cancer development (28). However, more studies are needed to confirm this view.

#### 3.1.3 Phases 1b/IIb trial (QUILT-2.005, NCT02138734)

The phase 1b trial with intravesical ALT-803 combined with intravesical BCG for the treatment of BCG-naïve NMIBC CIS with or without Ta or T1 disease has been completed (*N* = 9). The CR rates of the combined administration after 3 and 6 months of treatment were 78% and 88.9%, respectively. This combination achieved a 100% CR rate at 24 months, with no disease recurrence. However, BCG alone showed only a 50% response rate. Additionally, all participants had complete bladders and no disease recurrence 6 years after this combination treatment, suggesting that intravesical ALT-803 and BCG show long-term effective antitumor activity (29, 30). These data suggest that intravesical ALT-803 and BCG exhibit strong antitumor activity. However, as the number of patients treated with intravesical ALT-803 and BCG was small in the phase 1 trial, there was a need to conduct phases 2 and 3 clinical studies to validate the findings.

#### 3.1.4 Phases 2/3 trial (QUILT-3.032, NCT03022825)

Phase 2 and 3 trials for the combined use of intravesical ALT-803 and intravesical BCG for the treatment of BCGu NMIBC have been completed (*N* = 83 CIS, 77 papillary). The recommended dose of ALT-803 and BCG was 400 μg/instillation and 50 mg/instillation, respectively. This combination suppressed disease progression in patients with BCGu NMIBC. Approximately 71% (59/83) of patients with CIS treated with the combination achieved CR at any time. The median duration time of CR in these patients was 26.6 months. Approximately 96% of patients with CIS had a 24-month survival rate of not progressing to MIBC. Approximately 91% of patients with CIS who responded avoided cystectomy within 24 months. The patients in the papillary group achieved a disease-free survival rate of 57% in 12 months and 48% in 24 months with the combination treatment. Approximately 95% of the patients in the papillary group avoided cystectomy. Among the patients who did not avoid cystectomy, the median time to cystectomy was 5.1 months later in those who responded (*N* = 4, 12.9 months) than in those who did not respond (*N* = 8). The BCa-specific overall survival rate in 160 patients with BCGu NMIBC at 24 months was 99%. More than 90% of patients avoided cystectomy at 24 months of follow-up (31–34). The combination treatment also showed safety and tolerability in these patients, suggesting that intravesical ALT-803 combined with BCG has a good response rate and is safe. Additionally, ALT-803 in combination with BCG may induce a good response in patients with BCGu NMIBC in an immunosuppressive environment, such as in patients with low expression of CTLA4, CD39, and CCL2–CCR2 axis (35). Taken together, ALT-803 combined with BCG treatment has a better curative effect.

### 3.2 Pharmacokinetics

#### 3.2.1 Phase 1 trial (healthy human volunteers)

S.C. ALT-803 has a longer half-life than I.V. administration. Indeed ALT-803’s concentration remained above baseline at 3 days after a single S.C. dose and promoted the number of NK cells for at least 15 days. The half-life of S.C. ALT-803 is approximately 20 h, and its half-life and tissue exposure are, respectively, 20- and 2.3-fold higher than that of rhIL-15 (28), suggesting that the efficacy of ALT-803 is higher than that of rhIL-15 in humans. The serum ALT-803 levels peaked 4 h after S.C. ALT-803 in healthy human volunteers. The exposure and AUC of ALT-803 increased in a dose-dependent manner. The C_max_ of S.C. ALT-803 was found to range from 1,000 to 1,700 pg/ml. The PK profile of I.V. ALT-803, such as C_max_ and half-life, is higher (100-fold higher) and shorter (10-fold lower), respectively, than that of S.C. ALT-803. However, I.V. ALT-803 concentration easily induces fever and tachycardia compared with S.C. administration. The PK profiles and safety of ALT-803 in healthy human volunteers were similar to those in patients with cancer (28). Therefore, S.C. administration is preferable.

#### 3.2.2 Phases 1b/IIb trial (QUILT-2.005, NCT02138734)

In phases 1b/IIb trial, the PK analysis demonstrated serum ALT-803 in 30 min, 2 h, 4 h, and 24 h after intravesical ALT-803 administration (100, 200, and 400 µg per/instillation) in combination with intravesical BCG for the treatment of patients with BCG-naïve NMIBC (*N* = 9). ALT-803 was detected in only three patients in 24 h after intravesical ALT-803 and BCG administration. However, its level was below or near the detection limit, suggesting that intravesical ALT-803 administration is not metabolized by the whole body (30).

#### 3.2.3 Phases 2/3 trial (QUILT-3.032, NCT03022825)

The PK data of the phases 2/3 trial were similar to that of the phases 1b/IIb trial. No systemic ALT-803 was detected by intravesical ALT-803 and BCG administration in patients. Furthermore, the activity of ALT-803 was limited to the bladder and did not cause a systemic reaction (31, 32).

### 3.3 Toxicology

#### 3.3.1 Phase 1 trial (healthy human volunteers)

There were no serious adverse events (AEs) or grade ≥3 AEs reported after the administration of S.C. ALT-803 in 20 healthy human volunteers (11 male and nine female). The common side effects included injection site reactions (redness, hardness, pain, and itching), chills, fever, fatigue, hypoalbuminemia, abdominal pain, and headache. All the treatment-related side effects were resolved with supportive care. The degree of severity and frequency of AEs by S.C. ALT-903 was far less than that by I.V. administration in the clinical trial (NCT01885897), suggesting that S.C. administration may be more advantageous than I.V. administration (28).

#### 3.3.2 Phases 1b/IIb trial (QUILT-2.005, NCT02138734)

The AEs triggered by intravesical ALT-803 (100, 200, and 400 µg per instillation) in combination with intravesical BCG during the treatment of nine patients with NMIBC were less than grade 3 (30). The common AEs included fatigue (33%), urinary tract infection (22%), urinary tract pain (22%), urinary urgency (22%), urinary frequency (33%), headache (33%), hematuria (33%), dyspnea (22%), and hypertension (67%). ALT-803 has no dose-limiting toxicities and a maximum tolerated dose, suggesting that ALT-803 is well tolerated in patients. Serum ALT-803 was detected in 33.3% of patients (3/9). Furthermore, the serum did not contain any anti-ALT-803 antibodies in all the patients, suggesting that ALT-803 has no immunogenicity. Intravesical ALT-803 in combination with BCG in a non-dose-dependent manner increased urinary levels of IL-6, IL-2, IL-10, TNF-α, and IFN-γ but not of IL-4. This combination therapy did not alter the plasma cytokines, suggesting that intravesical ALT-803 did not cause any systemic cytokine storm (30).

#### 3.3.3 Phases 2/3 trial (QUILT 3.032, NCT03022825)

No serious treatment-related toxicities occurred in 99% of patients with BCGu NMIBC (160 patients) by intravesical ALT-803 combined with BCG treatment. The low AEs (grades 1 and 2) triggered by ALT-803 included dysuria (22%), hematuria (18%), pollakiuria (19%), urinary urgency (12%), and fatigue (16%); the occurrence of other low AEs was 7% or less. No immunogen-related side effects, serious AEs, and grade 4 or 5 AEs were observed (31–33). Thus, intravesical ALT-803 combined with BCG treatment is relatively well tolerated and safe (**Table 1**).

**Table 1:** The preclinical and clinical evidence uses of ALT-803 in NMIBC.

Property	Administration	Doses	Model	Effect		References
Pharmacology	S.C. ALT-803	0.2 mg/kg/week, 6 weeks	BBN-induced orthotopic NMIBC C57BL/6J mouse model	Reduced tumor burden	37%	(13)
	S.C. ALT-803 plus intravesical BCG	0.2 mg/kg/week plus 135 μg per/instillation/week, 6 weeks			44%	(13)
	Intravesical BCG	135 μg per/instillation/week, 6 weeks			28%	(13)
	Intravesical ALT-803	0.1 μg per/instillation/week, 6 weeks			28%	(13)
	Intravesical ALT-803 plus BCG	0.1 μg per/instillation/week plus 135 μg per/instillation/week, 6 weeks			44%	(13)
	Intravesical ALT-803	1 μg per/instillation/week, 6 weeks	BBN-induced orthotopic NMIBC SD rat model	Reduced tumor burden, tumor angiogenesis, tumor proliferation, bladder wall thickness	35%, 59%, 52%, 25%	(23)
	Intravesical ALT-803 plus BCG	1 μg per/instillation/week plus 135 mg per/instillation/week, 6 weeks			46%, 76%, 80%, 30%	(23)
	Intravesical BCG	135 mg per/instillation/week, 6 weeks			15%, 40%, 40%, 30%	(23)
	Intravesical ALT-803 plus BCG (phase 1b)	100, 200, and 400 μg per/instillation/week plus 50 mg per/instillation/week	Patients with BCG-naïve NMIBC (N = 9)	CR rate	78% (3 months), 88.9% (6 months), 100% (24 months)	(29, 30)
				Had complete bladders and no disease recurrence (6 years)		(29, 30)
	Intravesical ALT-803 plus BCG (phase 2/3)	400 μg per/instillation/week plus 50 mg per/instillation/week	Patients with BCGu NMIBC CIS (*N* = 83)	CR rate	71% (any time)	(31–34)
				Median duration time	26.6 months	(31–34)
				Not progressing to MIBC rate	96% (24 months)	(31–34)
				Avoided cystectomy rate	91% (24 months)	(31–34)
			Patients with BCGu NMIBC - Papillary (*N* = 77)	CR rate	57% (12 months), 48% (24 months)	(31–34)
				Median duration time to cystectomy (*N* = 4)	12.9 months	(31–34)
				Avoided cystectomy rate	95%	(31–34)
			Overall survival rate		99% (24 months)	(31–34)
			Overall avoided cystectomy rate		>90% (24 months)	(31–34)
Pharmacokinetics	S.C. ALT-803	0.2 mg/kg	B6 mice	*T*_1/2_	7.5 h	(22)
				*C*_max_	495 ng/ml at 16 h	(22)
		3–7 MBq		Tissue distribution	Spleen, lymph nodes, bone, liver, stomach, lung, heart, kidney, blood, brain, intestine, muscle, pancreas, thymus	(22)
		10 μg/kg, 20 μg/kg	Healthy humans	*T*_1/2_	~20 h	(28)
				*C*_max_	1,000–1,700 pg/ml at 4 h	(28)
	I.V. ALT-803	0.2 mg/kg	B6 mice	*T*_1/2_	7.71 h	(22)
				*C*_max_	3,926 ng/ml at 0.5 h	(22)
		3–7 MBq	B6 mice or C57BL/6 mice	Tissue distribution	Spleen, lymph nodes, bone, liver, stomach, lung, heart, kidney, blood, brain, intestine, muscle, pancreas, thymus, skin, tail	(21, 22)
		0.1 and 0.03 mg/kg	Cynomolgus monkeys	*T*_1/2_	7.2–8 h	(21)
				*C*_max_	30 nmol/L (0.1 mg/kg) 6 nmol/L (0.03 mg/kg)	(21)
	Intravesical ALT-803 plus BCG (phase 1b)	100, 200, and 400 μg per/instillation/week plus 50 mg per/instillation/week	Patients with BCG-naïve NMIBC (*N* = 9)	No	Systemic level of ALT-803	(30)
	Intravesical ALT-803 plus BCG (phases 2/3)	400 μg per/instillation/week plus 50 mg per/instillation/week	Patients with BCGu NMIBC (*N* = 160)	No	Systemic level of ALT-803	(31, 32)
Toxicology	S.C. ALT-803	1.0 mg/kg/week, 4 weeks	B6 mice	Decreased	ALP and creatinine	(22)
				Increased weight of the spleen, lymph node, and liver		(22)
		10 μg/kg, 20 μg/kg	Healthy humans (*N* = 20)	Increased injection site reactions (redness, hardness, pain, and itching), chills, fever, fatigue, hypoalbuminemia, abdominal pain, and headache		(28)
	I.V. ALT-803	0.1, 1 mg/kg/week, 4 weeks	C57BL/6 mice	Well tolerated and safe		(21)
		4.0 mg/kg/week, 4 weeks		Increased	Weight loss	(21)
					Death	(21)
					Pulmonary edema	(21)
					The weight of spleen, lymph node	(21)
		0.1 and 0.03 mg/kg/week, 4 weeks	Cynomolgus monkeys	Increased	Mild liver necrosis	(21)
		0.1 mg/kg/week, 4 weeks		Reduced	Albumin	(21)
	Intravesical ALT-803 alone or plus BCG	1 μg per/instillation/week plus 135 mg per/instillation/week, 6 weeks	BBN-induced orthotopic NMIBC SD rat model	No dose-limiting toxicity		(23)
	Intravesical ALT-803 plus BCG (phase 1b)	100, 200, and 400 μg per/instillation/week plus 50 mg per/instillation/week	Patients with BCG-naïve NMIBC (*N* = 9)	Increased fatigue, urinary tract infection, urinary tract pain, urinary urgency, urinary frequency, headache, hematuria, dyspnea, and hypertension		(30)
					<Grade 3	(30)
				No	DLTs and MTD	(30)
					Immunogen-related side effects	(30)
					Systemic cytokine storms	(30)
	Intravesical ALT-803 plus BCG (phases 2/3)	400 μg per/instillation/week plus 50 mg per/instillation/week	Patients with BCGu NMIBC (N = 160)	Increased	Dysuria, hematuria, pollakiuria, urinary urgency, and fatigue	(31–33)
				No	Grades 3–5	(31–33)
					Serious adverse events	(31–33)
					Immunogen-related side effects	(31–33)

S.C., subcutaneous; I.V., intravenous; NMIBC, non-muscle-invasive bladder cancer; BBN, N-butyl-N-(4-hydroxybutyl) nitrosamine; CR, complete response; BCGu, Bacillus Calmette–Guérin-unresponsive; CIS, carcinomas *in situ*; T_1/2_, half-life; C_max_, maximum serum concentration; DLTs, dose-limiting toxicities; MTD, maximum tolerated dose.

## 4 Translational potential of ALT-803: Phases 1–3 clinical comparison of pembrolizumab, OM, and ALT-803

As mentioned at the beginning, the FDA rejected the marketing application for OM for the treatment of BCGu NMIBC due to a lack of data comparing it with pembrolizumab. Therefore, we compared the clinical data of ALT-903, pembrolizumab, and OM. Pembrolizumab is an anti-PD1 monoclonal antibody (mAb). OM is a recombinant fusion protein that contains an anti-epithelial cell adhesion molecule antibody and *Pseudomonas* exotoxin A. Indeed OM also received an FDA fast-track designation in August 2018 and a priority review in February 2021 (36). The CR rates of I.V. pembrolizumab plus intravesical BCG after 3 months of treatment of nine patients with BCGu NMIBC was 56% in the phase 1 trial (37). The CR rate of intravesical OM after 3 months of treatment of 45 patients with BCGu NMIBC CIS was 40% in a phase 2 trial [37, 38], suggesting that the CR rate of OM after 3 months is lower than that of ALT-803 (78%) and pembrolizumab (56%). The phase 3 data of I.V. pembrolizumab for the treatment of 96 patients with BCGu NMIBC CIS have shown that the 3- and 12-month CRs are 40.6% and 18%, respectively (KEYNOTE-057). The phase 3 data of intravesical OM for the treatment of 89 patients with BCGu NMIBC CIS have shown that the 3- and 12-month CRs are 40% and 20%, respectively, suggesting that the CR rate of OM is similar to that of pembrolizumab but is lower than that of ALT-803 (71% at any time). The CR median duration of OM in these patients was 9.4 months, and 52% of the responders were free of the disease for 12 months; these values are lower than that observed for ALT-803 (26.6 months and 96% after 24 months) and pembrolizumab (16.2 months and 83% after 12 months). The 3-, 6-, 12-, and 24-month CR rates of intravesical OM in BCGu NMIBC with papillary disease (*N* = 38) were 71%, 58%, 50%, and 37%, respectively, and were lower than those observed with ALT-803 (57% and 48% at 12 and 24 months, respectively). The median duration of CR in these patients was 13.2 months, which is longer than that observed for ALT-803 (12.9 months). The median time (34 months) of responders to need cystectomy was 13.3 months later than that of nonresponders, and this value was longer than that for ALT-803, suggesting that the CR median time of OM in BCGu NMIBC with papillary disease is longer than that of ALT-803. Furthermore, 90% of responder patients avoided cystectomy at 24 months of follow-up, which is similar to that observed with ALT-803. The average overall survival rate of 126 patients with BCGu NMIBC after OM treatment at 24 months was 96%, which is lower than that observed for ALT-803 (99%) and pembrolizumab (98%). The therapeutic dose of OM is 30 mg at two times per week and is lower than that of pembrolizumab (200 mg, three times per week) but higher than that of ALT-803 (400 μg, one time per week). These results suggest that the clinical efficacy of OM may be similar to that of pembrolizumab but is lower than that of ALT-803.

Pembrolizumab and OM are well tolerated and safe. The majority of AEs of pembrolizumab are grades 1 and 2 and include fatigue (10.9%), pruritus (10.9%), and diarrhea (10.9%) (38, 39). However, 12.9% of these patients develop severe AEs, including immune-related AEs (21.8%), grades 3 and 4 (3.0%), and these numbers are higher than that observed with ALT-803. The majority of AEs of OM are also grades 1 and 2. However, 2% of these patients develop severe AEs, including pyrexia (grade 2), acute kidney injury (grade 3), cholestatic hepatitis (grade 4), and renal failure (grade 5) (40). These observations suggest that OM is safer than pembrolizumab, while ALT-803 is safer than OM (as ALT-803 shows no grade 3–5 AEs). Taken together, the clinical efficacy and safety of OM may be lower than that of ALT-803 except for the median duration time of responders in papillary disease. The clinical efficacy of OM may be similar to that of pembrolizumab, but the safety of OM may be higher than that of pembrolizumab (**Table 2**). Thus, ALT-803 has a strong clinical application value. However, more studies are needed to further validate its clinical use.

**Table 2:** Phases 1–3 clinical comparison of ALT-803, pembrolizumab, and oportuzumab monatox (OM) for the treatment of Bacillus Calmette–Guérin-unresponsive NMIBC.

Study	Property	ALT-803		Pembrolizumab	OM	
Diseases		NMBIC CIS	NMBIC papillary	NMBIC CIS	NMBIC CIS	NMBIC papillary
Phases 1 and 2	Number	9		9	45	
	CR rates	78% (3 months)		56% (3 months)	40% (3 months)	
Phases 2 and 3	Number	83	77	96	89	38
	Administration	Intravesical	Intravesical	I.V.	Intravesical	Intravesical
	Therapeutic dose	400 μg, one time/week	400 μg, one time/week	200 mg, three times/week	30 mg, two times/week	30 mg, two times/week
	CR rates	71% (anytime)	57% (12 months), 48% (24 months)	40.6% (3 months), 18% (12 months)	40% (3 months), 20% (12 months)	71% (3 months), 58% (6 months), 50% (12 months), 37% (24 months)
	CR duration time	26.6 months	12.9 months	16.2 months	9.4 months	13.2 months
	No disease rate	96% (24 months)		83% (12 months)	52% (12 months)	
	Avoided cystectomy time		12.9 months			34 months
	Avoided cystectomy rate		90%			90%
	Overall survival	99%		98%	96%	
	AEs grades 1 and 2	Dysuria (22%), hematuria (18%), pollakiuria (19%), urinary urgency (12%), fatigue (16%)		Fatigue (10.9%), pruritus (10.9%), diarrhea (10.9%)	52%		
	Severe AEs	No grades 3–5, serious AEs, immunogen-related side effects		12.9%, including immune-related AEs (21.8%), grades 3 and 4 (3.0%)	2%, including pyrexia, acute kidney injury, cholestatic hepatitis, renal failure	

I.V., intravenous; NMIBC, non-muscle-invasive bladder cancer; CIS, carcinomas *in situ*; CR, complete response; AEs, adverse events.

## 5 Future perspectives

**Figure 2:**
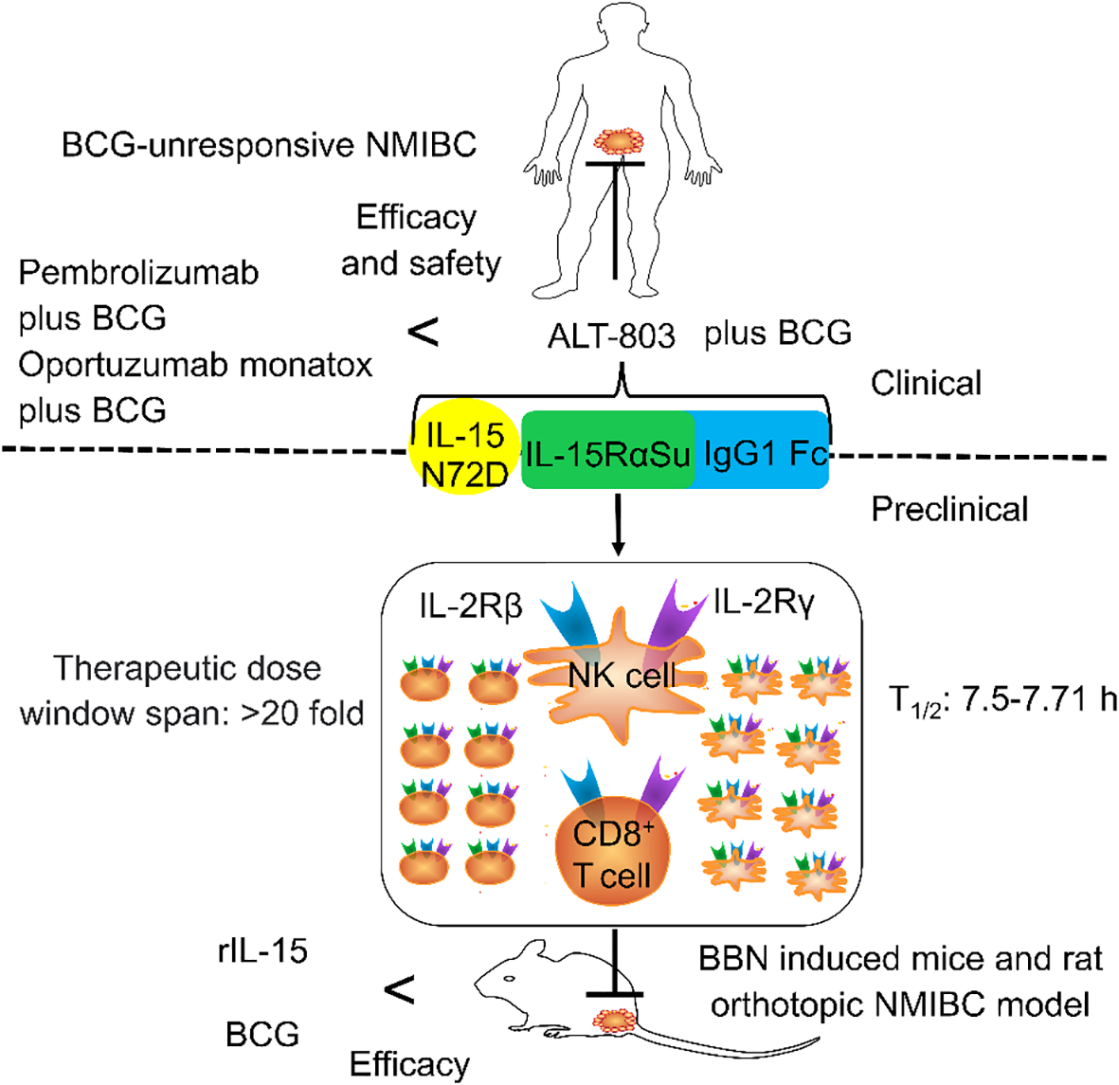
The general structure of ALT-803 and its role in non-muscle-invasive bladder cancer (NMIBC). ALT-803 contains IL-15 N72D, IL-15RαSu, and IgG1 Fc. IL-15 N72D promotes the proliferation and activation of NK and T cells by enhancing the activity of binding to IL-2Rβ. IL-15RαSu can selectively transexpress the IL-2Rβγ chain of NK and CD8+ T cells without expanding Tregs. IgG1 Fc not only prolonged the half-life but also increased the circulation and distribution of lymphocytes. The preclinical efficacy and safety of ALT-803 were higher than that of Bacillus Calmette–Guérin (BCG) and rIL-15. The clinical efficacy and the safety of ALT-803 plus BCG for the treatment of patients with BCG-unresponsive NMIBC were higher than that of pembrolizumab plus BCG and oportuzumab monatox plus BCG.

ALT-803 is a promising drug for the treatment of NMIBC as it not only reduces tumor burden but also enhances the efficacy of BCG (**Figure 2**). However, several interesting and critical tasks remain to be explored, namely:

1. The preclinical models of BCa include *in vitro* 2D models, such as cell lines from human, mouse, rat, dog, and conditionally reprogrammed cell cultures, *in vitro* 3D models, such as organoids and three-dimensional (3D) printing, *in vivo* models, such as carcinogen (such as BBN, amines, anthracenes, formamide FANFT)-induced model, genetically engineered mouse models, patient-derived xenograft models, and humanized models. However, the efficacy of ALT-803 has only been revealed in the BBN-induced rat and mouse models (41).
2. S.C. ALT-803 in combination with intravesical BCG was more effective than intravesical ALT-803 and BCG in the preclinical trial. The PK and the safety of S.C. ALT-803 are similar to that of intravesical administration in the preclinical and phase 1 trial. However, the efficacy of S.C. ALT-803 in combination with intravesical BCG for the treatment of NMIBC has not been investigated.
3. The biomarkers for predicting the response to ALT-803 remain unclear.
4. I.V. ALT-803 at 1.0 mg/kg/week is well tolerated and shows strong immune cell stimulation in C57BL/6 mice. Interestingly, I.V. ALT-803 at 0.05 mg/kg exhibits a strong antitumor effect in the 5T33 myeloma mouse model, suggesting that the therapeutic dose window for ALT-803 spans over a 20-fold range (21). However, the dose window of ALT-803 for the treatment of NMIBC still needs to be investigated.
5. I.V. ALT-803 takes less time to achieve efficacy than S.C. and intravesical administration, suggesting that I.V. administration is more appropriate for acute complications. However, some side effects should be noted, such as fever and tachycardia. Therefore, further studies are needed to verify the efficacy of I.V. ALT-803.
6. Toxicology indicated that ALT-803 is relatively safe. However, S.C. ALT-803 reduced the blood ALP in mice. I.V. ALT-803 promoted pulmonary edema and even death in mice. I.V. ALT-803 induced liver necrosis and reduced serum albumin in monkeys. Therefore, clinical attention should be paid to these side effects even though they have not been observed so far.
7. The systemic immune response and the efficacy of intradermal BCG before intravesical administration for the treatment of NMIBC are being examined in a phase 3 trial (NCT03091660). It is speculated that intradermal BCG injection before intravesical BCG injection may improve the BCG relapse-free survival rates (42). However, the final results are not yet available. The efficacy and the safety of this therapy in combination with S.C. or intravesical ALT-803 also remain unclear.
8. ALT-803 enhanced the antibody-dependent cell-mediated cytotoxicity (ADCC) capacity mediated by avelumab (anti-PD-L1 monoclonal antibody), which is being developed for the treatment of bladder carcinomas in a phase 2 trial (43). However, the efficacy of ALT-803 in combination with avelumab for the treatment of NMIBC has not been investigated.
9. M7824 (MSB0011359C) is a bifunctional fusion protein that contains anti-PD-L1 monoclonal antibody (the structure is similar to that of avelumab) and TGFβR2. ALT-803 also enhanced the ADCC capacity mediated by M7824. The tumor cell lysis activity of ALT-803 was similar to that of M7824 in H441 (lung carcinoma), HCC4006 (lung carcinoma), MDA-MB-231 (breast carcinoma), and CaSki (cervical carcinoma) cells (43). Phase 1 trials for the treatment of advanced non-small cell lung cancer and advanced solid tumors using M7824 have been completed (44, 45). M7824 promotes human urothelial carcinoma cell lysis by increasing the CXCL11, TRAIL, and CD8^+^ T cell activity in HTB-1/4/5 cells, suggesting that M7824 may also promote tumor cell lysis in NMIBC (46). However, the efficacy of ALT-803 in combination with M7824 for the treatment of NMIBC has not been investigated. Whether ALT-803 is better than M7824 for the treatment of NMIBC has also not been investigated.
10. Intravesical liposomal IL-15 reduced the tumor burden by 52% by activating CD8^+^ T cell in MB49-induced mouse orthotopic BCa models. Upon liposomal IL-15 treatment, these mice also displayed immunologic memory that resisted the growth of the BCa, suggesting that the efficacy of liposomal IL-15 is higher than that of BCG (47). However, the efficacy of ALT-803 in this model has not been investigated. It is not clear whether ALT-803 is better than liposomal IL-15 even though their targets are the same.

Finally, although the IL-15 superagonist ALT-803 showed high safety in preclinical and clinical studies, one needs to be wary. IL-15 can be considered a double-edged sword, as it stimulates the wound-healing migration and invasion of bladder cancer 5637 cells by stimulating the p21WAF1/ERK1/2/NF-κB/MMP-9 signaling pathway. Furthermore, IL-15 promotes the growth, migration, and invasion of tumor cells, including prostate cancer cells, colon cancer cells, and trophoblast cells (48). ALT-803 is also a double-edged sword in haplo-NK therapy (49). Indeed IL-15 has antitumor effects based on its ability to enhance the proliferation and anti-apoptotic effect of immune cells. Thus, we should be aware of the risks posed by super-excited IL-15.

## 6 Conclusion

In the present review, we summarize and discuss the translational potential of ALT803 for the treatment of NMIBC from the perspective of pharmacology, pharmacokinetics, and toxicology based on preclinical and clinical evidence. ALT-803 increases the proliferation and activation of NK cells and CD8^+^ T cells by serving as a superagonist of IL-15, leading to a reduction of tumor burden. ALT-803 alone or in combination with BCG exhibits good tolerability, pharmacology, pharmacodynamics, pharmacokinetics, and safety. The phases 2 and 3 data of ALT-803 are better than that of BCG, valrubicin, pembrolizumab, and OM. It is expected that ALT-803 will be on the market soon and become the best therapy for the treatment of NMIBC in the near future. OM may also be marketed. However, no robust head-to-head preclinical and clinical trials have been conducted to compare ALT-803 with valrubicin, pembrolizumab, and OM. More further studies are needed. Overall, we sincerely hope that many more scientists will focus on ALT-803 and its target IL-15 to develop more new drugs that can be used in the treatment of NMIBC.

## Author contributions

WC and NL conducted the literature review, drafted the initial manuscript, designed the figure, and approved the final manuscript as submitted. YY and MZ designed the figure, reviewed the initial manuscript, and approved the final manuscript as submitted. XH and WH reviewed the initial manuscript and approved the final manuscript as submitted. SW and CW conceptualized the study, reviewed the initial manuscript, and approved the final manuscript as submitted. BH and DX conceptualized and supervised the study, obtained funding, conducted the literature review, drafted the initial manuscript, designed the figure, and approved the final manuscript as submitted. All authors contributed to the article and approved the submitted version.

## Funding

The authors are grateful for the financial support provided by the Qingdao Major Scientific and Technological Project for Distinguished Scholars (20170103), the Laoshan Major Scientific and Technological Project for Distinguished Scholars (20181030), and the Natural Science Foundation of Shandong Province (ZR2020MH369, ZR2020MH242).

## Acknowledgments

We thank colleagues in Dr. DX’s laboratory for their technical help and stimulating discussions during this investigation. We thank Bullet Edits Limited for linguistic editing and proofreading of the manuscript.

## Conflict of interest

The authors declare that the research was conducted in the absence of any commercial or financial relationships that could be construed as a potential conflict of interest.

## Publisher’s note

All claims expressed in this article are solely those of the authors and do not necessarily represent those of their affiliated organizations, or those of the publisher, the editors and the reviewers. Any product that may be evaluated in this article, or claim that may be made by its manufacturer, is not guaranteed or endorsed by the publisher.

## Glossary

**Table:**

BCa	Bladder cancer
NMIBC	Non-muscle-invasive bladder cancer
BCG	Bacillus Calmette–Guérin
BCGu	BCG-unresponsive
CR	Complete remission
OM	Oportuzumab monatox
PDUFA	Prescription Drug User Fee Act
MIBC	Muscle-invasive bladder cancer
T1	Lamina propria
CIS	Carcinomas *in situ*
Ta	Papillary noninvasive carcinomas
FDA	United States Food and Drug Administration
NK	Natural killer
IL-2	Interleukin-2
INF-γ	Interferon-gamma
FTD	Fast track designation
BTD	Breakthrough designation status
S.C.	Subcutaneous
I.V.	Intravenous
GzB	Granzyme B
rIL-15	Recombinant IL-15
BBN	N-Butyl-N-(4-hydroxybutyl) nitrosamine
C_max_	Maximum serum concentration
B6	C57BL/6NHsd
CM	Central memory
EM	Effector memory
ALP	Alkaline phosphatase
PBMCs	Peripheral blood mononuclear cells
DFS	Disease-free survival
PK	Pharmacokinetic
AEs	Adverse events
DLTs	Dose-limiting toxicities
MTD	Maximum tolerated dose
mAb	Monoclonal antibody
EpCAM	Epithelial cell adhesion molecule
ETA	*Pseudomonas* exotoxin A
CRC	Conditionally reprogrammed cell
3D	Three-dimensional
GEMM	Genetically engineered mouse models
PDX	Patient-derived xenograft
ADCC	Antibody-dependent cell-mediated cytotoxicity
NSCLC	Non-small cell lung cancer
